# The impact of training non-physician clinicians in Malawi on maternal and perinatal mortality: a cluster randomised controlled evaluation of the enhancing training and appropriate technologies for mothers and babies in Africa (ETATMBA) project

**DOI:** 10.1186/1471-2393-12-116

**Published:** 2012-10-25

**Authors:** David Ellard, Doug Simkiss, Siobhan Quenby, David Davies, Ngianga-bakwin Kandala, Francis Kamwendo, Chisale Mhango, Joseph Paul O’Hare

**Affiliations:** 1 Warwick Clinical Trials Unit, Division of Health Sciences, Warwick Medical School, The University of Warwick, Coventry CV4 7AL, UK; 2 Division Mental Health & Wellbeing, Warwick Medical School, The University of Warwick, Coventry, CV4 7AL, UK; 3 Division of Reproductive Health, Warwick Medical School, The University of Warwick, Coventry, CV4 7AL, UK; 4 Educational Development & Research Team, Warwick Medical School, The University of Warwick, Coventry, CV4 7AL, UK; 5 Division of Health Sciences, Warwick Medical School, The University of Warwick, Coventry, CV4 7AL, UK; 6 Obstetrics and Gynaecology Department, Malawi University, College of Medicine, P/B 360, Blantyre, Chichiri, Malawi; 7 Mwaiwathu Pvt. Hospital, P. O. Box 3067, Blantyre, Malawi; 8 Division of Metabolic & Vascular Health, Warwick Medical School, The University of Warwick, Coventry, CV4 7AL, UK

## Abstract

**Background:**

Maternal mortality in much of sub-Saharan Africa is very high whereas there has been a steady decline in over the past 60 years in Europe. Perinatal mortality is 12 times higher than maternal mortality accounting for about 7 million neonatal deaths; many of these in sub-Saharan countries. Many of these deaths are preventable. Countries, like Malawi, do not have the resources nor highly trained medical specialists using complex technologies within their healthcare system. Much of the burden falls on healthcare staff other than doctors including non-physician clinicians (NPCs) such as clinical officers, midwives and community health-workers. The aim of this trial is to evaluate a project which is training NPCs as advanced leaders by providing them with skills and knowledge in advanced neonatal and obstetric care. Training that will hopefully be cascaded to their colleagues (other NPCs, midwives, nurses).

**Methods/design:**

This is a cluster randomised controlled trial with the unit of randomisation being the 14 districts of central and northern Malawi (one large district was divided into two giving an overall total of 15). Eight districts will be randomly allocated the intervention. Within these eight districts 50 NPCs will be selected and will be enrolled on the training programme (the intervention). Primary outcome will be maternal and perinatal (defined as until discharge from health facility) mortality. Data will be harvested from all facilities in both intervention and control districts for the lifetime of the project (3–4 years) and comparisons made. In addition a process evaluation using both quantitative and qualitative (e.g. interviews) will be undertaken to evaluate the intervention implementation.

**Discussion:**

Education and training of NPCs is a key to improving healthcare for mothers and babies in countries like Malawi. Some of the challenges faced are discussed as are the potential limitations. It is hoped that the findings from this trial will lead to a sustainable improvement in healthcare and workforce development and training.

**Trial registration:**

ISRCTN63294155

## Background

Maternal mortality and morbidity associated with pregnancy remain major challenges to improving health in Africa. Prior to 2005 it was reported that six hundred thousand women die every year as a result of complications from pregnancy and childbirth [1]. The World Health Organisation reported in 2008 that there were 358,000 maternal deaths globally and while there have been some improvements since [2], most of these deaths are preventable. Human resources and the effective service delivery of appropriate sustainable technologies have been identified as key areas that need support if this global inequity in health is to be improved. The Millennium Development Goals 4 and 5 of reducing maternal and perinatal mortality can only be achieved by developing and evaluating innovative transferable and sustainable solutions through collaboration between African and International partnerships.

Maternal mortality in most of sub-Saharan Africa remains obstinately high [3]. In Malawi, for example, the maternal mortality ratio is 675/100,000 whereas in the UK it is 13/100,000 [3-5]. Whereas there has been a steady decline in maternal mortality in Europe over the past 60 years, in Africa even long periods of stability and increases in health spending have had little effect in many countries [5]. The UN has set a target for maternal case fatality rate of less than 1%. Many women in rural and urban communities in Africa give birth without any trained assistance for their pregnancy and childbirth as less than 50% of women in low-income countries are attended by skilled health personnel at birth, yet life-threatening complications that require emergency care will arise for around 15% [6]. Perinatal mortality is 12 times higher than maternal mortality and accounts for seven million deaths: about three million babies are stillborn and four million die in the neonatal period. Much of this loss is preventable [3,6,7]. The major causes of the almost four million neonatal deaths in low-income countries in or around the first week of life are infection, pre-term birth and asphyxia [6].

Models of healthcare, which have developed in Europe, based on highly trained medical specialists using complex technology, are unlikely to be a practical way forward or sustainable in sub-Saharan Africa. There is evidence to support a different model of service provision in Africa, whereby the relatively scarce resource of medical obstetric specialists are focused to train and support a service mainly provided by healthcare staff other than doctors, i.e. non-physician clinicians (NPCs) such as assistant medical officers, clinical officers, midwives and outreach community health-workers. In this model, the medically trained specialist obstetricians, mainly operating in large centres and capital cities, can focus their attention on management of difficult clinical cases and on providing support, leadership and training for NPCs. Many women in rural and urban communities in Africa give birth without any trained assistance for their pregnancy and childbirth. In sub-Saharan Africa, due to training and retention difficulties, there are only 5 doctors per 100,000 people [1] and in Malawi it is even worse with only 2 per 100.000 [8]. Programmes of training for health-workers to provide safe outreach community healthcare are being developed but these need to be systematic, transferable, and able to be scaled up to meet the needs of these women across Africa. A health delivery model of non-physician clinicians (NPCs) with support and supervision of the physician specialist obstetricians would be an affordable and sustainable system for these communities.

Work has been done to assess the efficiency of training NPCs (assistant medical officers, clinical officers and specialist midwives) in the skills of clinical decision-making and surgical intervention [1,7]. Training skilled attendants to prevent, detect and manage major obstetric complications, including undertaking emergency caesarean surgery in complicated deliveries is arguably the single most important factor in preventing maternal deaths and protecting the human rights of women [1,7,9,10]. To be effective NPCs need appropriate equipment, drugs and technology essential for managing obstetric complications in rural or deprived communities.

Task shifting from physicians to non-physicians appears to be both safe and effective in countries that have organised and supported the extension of their maternal care in this way [1,9-14]. Major surveys consistently show that extra training and support can achieve task shifting and improve maternal and foetal mortality and morbidity in the areas where these schemes have been piloted [9,11,13]. Most of the maternal population in sub-Saharan Africa lives in rural areas and for these women there remains major challenges to effective maternal care. Solutions must include outreach of effective care to this population. In addition to lack of available trained manpower, factors that have been identified as contributing to the higher maternal and perinatal mortality, include poor availability of relatively cheap drugs and simple technologies for managing post-partum haemorrhage (PPH); shortages of immediately available blood; lack of access to senior advice at all times; access to facilities and staff for emergency Caesarean sections; and delays and inadequacies in the safe transport to hospital when complications arise. There can also be a problem recognising complications early enough for effective action, (for example; breech, transverse lie, placenta praevia, pre-eclampsia and anaemia). Early detection of these could be improved with training and simple technologies. It is estimated that 75% of maternal deaths and more than 60% of perinatal deaths are caused by 8 major conditions. For the mother the 5 major killers are post-partum haemorrhage, sepsis, hypertensive disorders of pregnancy, obstructed labour, and unsafe abortion and the 3 major causes of perinatal mortality are low birth-weight, birth asphyxia, and infection [11,15].

### Rationale/justification for the research project

In Malawi, training and deployment of NPCs can be traced as early as 1875 when Dr Robert Laws started on the job training of Medical Orderlies and Medical Assistants. The Government introduced formal training of Clinical Officers in 1976 and this cadre is a major human resource for health in Malawi as far as clinical services are concerned; performing surgical procedures, giving anaesthetics and providing medical care. NPCs have been established health providers in Malawi for a long time, yet lack a clear career pathway [16,17]. Currently there is disparity between Malawi’s service coverage figures and maternal and child mortality. For example, coverage for skilled attendance and childbirth is 72% (Malawi DHS 2010) and contraceptive prevalence is 42% and yet maternal and child mortalities are very high. It is hoped that by providing NPCs with advanced leadership and skills training (the intervention) we will have an impact on maternal and perinatal morbidity and mortality) and help to strengthen the position of mid-level providers to expand cost-effective, quality services to under-serviced areas and thereby improve equitable access to care.

## Aims of the study

### Broad aims

The main aims of this study are to evaluate the impact on health outcomes (e.g. maternal and perinatal morbidity and mortality) of the intervention comparing intervention districts with control districts, and how well (or poorly) the intervention was implemented including how acceptable it was to all stakeholders.

### Specific aims

To explore changes in maternal and perinatal mortality outcomes comparing intervention districts with controls.

The primary outcome is perinatal mortality (pragmatically defined in this study as fresh stillbirths and neonatal deaths before discharge from the health care facility)

Secondary outcomes include:

• Maternal death rates (case specific);

• Recorded data (e.g. still births, Post-Partum Haemorrhage, Caesarean Section, Eclampsia, Sepsis, Neonatal resuscitation);

• Availability of resources (e.g. are drugs/blood available);

• Use of available resources (e.g. are drugs being used).

Alongside this we plan to carry out a process evaluation of the implementation of the intervention to inform future implementation of interventions like these or to develop it further for the future. The process evaluation will include outcomes which will explore how or why the intervention was either effective or not effective. Including:

• What was done and by whom;

• Challenges faced;

Acceptability;

• Sustainability.

Process evaluations particularly help researchers understand the causal pathways by which complex interventions might work and sometimes to interpret equivocal results. The shift towards greater evidence-based-practice means there is a greater need to know why an intervention works and, if it does not, why not. Process evaluation can facilitate this understanding and should be incorporated into the evaluation of health promoting interventions/programmes [18].

## Methods

### Design

The study is a cluster randomised controlled trial.

### Study place

The study will be conducted in districts within the central and northern regions of Malawi. There are a total of 14 districts in these regions which will be randomised to either intervention or control (Dedza, Dowa, Kasungu, Lilongwe, Mchinji, Nkhotakota, Ntcheu, Ntchisi, Salima, Chitipa, Karonga, Mzimba, Nkhata Bay, and Rumphi). A pragmatic decision was made that as Lilongwe is such a large district it would be divided into two with one half randomised to intervention and the other to control making a total of 15 districts. Stratified randomisation of the districts, to ensure the two groups are comparable will be carried out by a statistician at the University of Warwick, UK who will then inform the study team of the outcome. There will be 8 intervention districts and 7 controls. Control districts will be offered the intervention at the end of the trial as part of a randomised waiting-list design.

### Research team

The primary data collection team will consist of a research assistant based out of the College of Medicine (in Blantyre, Malawi) and a selection of trained clinical officers from each of the intervention districts (1 or 2 from each district). These clinical officers will be provided with basic training in research methods and full training collecting the quantitative data from their own and an adjacent control district by the research team at the University of Warwick. The Malawi team will be supported locally by colleagues within the Ministry of Health and the College of Medicine and by visits from the UK team; as well as regular electronic/mobile contact. The College of Medicine research assistant will visit all districts and carry out checks on data recording accuracy as well as providing a point of contact and support.

### Study population

Within the eight intervention districts approximately 50 NPCs will receive advanced leadership and skills training (the intervention). In each district randomised to the intervention between 3 and 8 NPCs (the number depends on size of district but a minimum is 3 in one district with the overall total not exceeding 50). The research assistants will invite the ‘trained’ NPCs to be involved in the evaluation. Their involvement will be related to the process evaluation and will include interviews and recording their project based activities. District medical and nursing officers in the intervention districts will be invited to be interviewed about the districts involvement in the intervention and at follow-ups how the intervention has worked/fitted in to the hospital routine. As part of the intervention the trained NPCs are expected to cascade the training they have received to others within their districts (e.g. other NPCs or midwives). The research assistant will identify a number of these people from the NPC’s records and they will be approached and interviewed about the training they received and how they have been able or indeed unable to implement what they have been taught. All participants will be provided with information about the study and asked to provide written informed consent.

### Study period

Anticipated start date is 01/11/2011 with an anticipated end date of 28/02/2014

### Sample size

The primary sample for the RCT is the fifteen districts, the health facilities within them and 50 participating NPCs from the 8 intervention districts.

#### ***Justification of sample size***

##### Power calculation

The project’s primary outcome measure is the proportion of live-born infants who died in the hospital or health facility in the early neonatal period, i-e, from birth to the day of discharge from facility. Other outcome measures of interest considered are the comparisons of proportions of fresh stillbirths. We computed a sample size for proportion in an unmatched study with 80% power, a one sided alpha of 0.05, and an Inter Class Correlation of 0.0025. The current neonatal mortality rate in Malawi: is 30 per 1000 live births [19] and assuming a minimum number of clusters of 14 in our sampled districts, the study was powered to detect a 20% difference between the two birth cohorts (intervention and control) in the proportion of live-born neonates delivered by NPCs or staff trained by them) surviving to hospital discharge.

With the allocation of 7 districts per arm with an estimated 700 births per NPC (or staff trained by them), 1028 births per study arm per district would provide sufficient power for a total of 2056 neonates per district. That is, a decline from 30 per 1000 live births to 24 per 1000 live births, rate ratio 0.20.

### Inclusion criteria

Only NPCs providing emergency obstetric and neonatal care (EmONC) from the 7 randomised intervention districts in central and northern region who have received the intervention training will be invited and those who will give their informed consent will be included in the project or:

The district medical or nursing officers (in intervention districts) or

NPCs, midwives or nursing staff who have been trained by one of the intervention NPCs

### ***Data collection***

#### ***Quantitative data collection***

Primary data will be extracted from the maternity log (Malawi Ministry of Health Maternity Register, Version 2 (July 2008)) at the district hospital and rural hospitals in each district by the research assistants monitored by the local and UK team. Other facilities within the district (e.g. health Centres) also complete the same maternity log book from which summary data is returned to the district hospital on a monthly basis. These data will also be gathered by the researchers and the combined data will make up a complete picture of the districts. Data will be collected at three points in time. Baseline data will be collected on cases (from the maternity logs and summary logs) for the 12 months prior to the date the training was delivered with two follow-ups at 12 monthly intervals. A paper case report form (CRF) will be produced to facilitate data collection. Data will then be transferred to an MS Excel spread sheet for transfer to the study database.

It is planned that our primary data will be that collected at source (i.e. each district). However, for the purposes of comparisons national data from national databases will also be sourced via our collaboration with the Malawian Ministry of Health for the same time periods.

Within the intervention districts at the three time points (baseline, 12 and 24 months) the research assistants will approach the consenting NPCs (primarily to interview them, described below) but also to gather information about project related activities (e.g. who they have trained, when they did this, how many training session done, etc.).

For process evaluation purposes training registers, adherence to training procedures (during the project period), knowledge scores and training feedback will also be collected and collated from the intervention team. No identifiable data will be recorded

#### ***Qualitative data collection***

In the intervention districts interviews will be carried out at each of the three time points with consenting NPCs (who have received the intervention training). These will be semi-structured interviews about their experiences relating to the project and its impact on practice. District medical and nursing officers in the intervention districts will be invited to be interviewed about the districts involvement in the intervention and at follow-ups how the intervention has worked/fitted in to the hospital routine. As part of the intervention the trained NPCs are expected to cascade the training they have received to others within their districts (e.g. other NPCs or midwives). The research assistants will identify a number of these people from the NPC’s records and they will be approached and interviewed about the training they received and how they have been able or indeed unable to implement what they have been taught. Below is more detail about the interviews. In addition to those already noted we plan to interview the intervention facilitators towards the end of the project again to enhance the process evaluation.

#### ***In-depth interviews with the trained clinical officers***

These interviews will mainly capture what the clinical officers that have been trained think about the training sessions. This will include feelings about the facilitators of the training, the content of the training, the training materials, the training period, and their expectations about the training. In addition participants will also be asked how the training has been beneficial to them, whether it has really improved their skills and also how easy or difficult it has been for them to train to fellow service providers. Plus included in this interview will be:

#### ***Critical incident interviews***

These will be specifically designed to capture the challenges and obstacles that the trained clinical officers met and affected their performance after acquiring the required skills. The interviews will also capture how the trained clinical officers overcame the challenges. This will involve the clinical officers narrating a specific incident or situation e.g. managing a woman with PPH, the obstacle to doing that and how it was overcome. 50 critical incident interviews will be conducted, which means all clinical officers that have been trained in the ETATMBA program will participate.

#### ***In-depth interviews with service providers trained by the trainee clinical officers***

These interviews will focus on how well the trainee clinical officers have implemented the training program to their fellow service providers in their respective districts. Selected service providers that have been trained by the trainee clinical officers will be interviewed on how well they have received the training and on how beneficial they think the training given by trainee clinical officers is to them.

#### ***In-depth interviews with the district medical officer and the nursing officer***

Key personnel in hospitals from which the clinical officers come from will also be interviewed to find out how the Clinical officers have implemented the training program in their respective district hospitals. In addition the key personnel will also give their views on whether they have been changes in the competence and performance of the trained clinical officers after receiving the training as well as on the service providers that have been trained by the clinical officers.

#### ***In-depth interviews with facilitators of the training program***

These interviews will capture facilitators’ feelings about the training. This will include their opinions on the content, training materials and period of training. In addition the facilitators will also be asked their general feelings about their participants and how easy or difficult it has been to train them.

Researchers will, during visits, make field notes noting their own activities (e.g. time taken collecting data, interviews arranged, interviews cancelled, challenges) but also observations made during the visit (things they see related to the project). These notes will be collated and analysed as a source of information for the process evaluation.

### Procedure

Randomisation of the districts will take place to identify the eight intervention districts. The intervention will then be offered to NPCs within these districts. The intervention team will employ a selection process to achieve appropriate participants in each district. However, this recruitment and selection process is not part of this research study.

Once district allocation is known letters will be sent from the Malawi Health Ministry to the District Medical Officers informing them about the project and their allocation to it. These letters inform the district officers that the research team will be contacting them to make arrangements to start collecting data.

The intervention training (described in more detail below) is delivered in cohorts away from the workplace. Within a couple of weeks of returning researchers from the team will visit the district. During this first visit they will gather the baseline quantitative data (described above) from district maternity records. They will also approach the trained NPCs and ask if they were willing to be involved in the evaluation. If so written informed consent will be obtained. Mutually agreeable times for interviews will be arranged being aware of the demands on time of their role.

During interviews the researcher, will review the registers of training sessions run by the trainee (recorded in their log books), randomly select two of these people and arrange short interviews with them (firstly obtaining written informed consent). They will also arrange to interview the district medical officer and the district nursing officer.

Data will be gathered at three points in time; baseline and two follow-ups at 12-month intervals. Written informed consent will only be obtained from new interviewees at follow-up, those consented earlier will be approached and retain the right to decline.

Control districts will be visited by researchers at similar time points to the intervention (baseline, 12-months & 24-months). Data will be gathered from the maternity logs and district maternity summary sheets as in the experimental districts. However, no NPCs will be interviewed in these hospitals.

### Intervention

Enhancing Training and Appropriate Technologies for Mothers and Babies in Africa (ETATMBA) is European Commission Framework 7 funded collaboration between the University of Malawi, the Malawi Ministry of Health, the University of Warwick (UK), the Karolinska Institute (Sweden), Ifakara Health Institute in Tanzania and GE Healthcare (UK). The project is working in Tanzania and Malawi but this RCT will only evaluate the impact of the intervention delivered in Malawi.

#### ***The training package***

The intervention is an 18 month programme of skills training and practice for NPCs in specific obstetric, neonatal and leadership skills delivered over three week-long intensive training modules with follow-up independent and workplace learning in year 1 followed by a six-month in-service training period to apply enhanced teaching, training and clinical audit.

Module 1 will consist of in depth theoretical and practical review of the prevention and management of the five major causes of  maternal mortality and the three most common causes of neonatal death; birth asphyxia, low birth weight and infection. Module 2 will deal with leadership, values-based practice and clinical service improvement, and Module 3 will deal with pre-term labour. The control districts will continue with their usual EmONC services.

Assessment of knowledge, competence and performance will be examined at the start of the programme and satisfaction, assessment of knowledge, competence and performance will be examined at the end. Trainees will have to successfully complete and pass a number of tasks (including audits, training others, reflective practice) Trainees will be asked to complete a short feedback questionnaire at the end of each days training noting what they feel they have learned and how valuable the training was to them.

The training programme addresses the major causes of maternal and perinatal mortality, how to teach, and research and leadership skills.

Practical and operative skills in the intervention districts will be supported by a specialist registrar in obstetrics working for a period of four weeks with NPCs to reinforce training, and rotating over the first year to all intervention hospitals. This on the job supervision and support will be supplemented by mobile and electronic communication between trainees and specialist consultants.

### Data analysis

#### ***Primary outcomes***

Data from the district maternity registers will be analysed in an interrupted time-series. The full data set will consist of 3 full years of data (12-months prior to the start of the project and 24 months of the project). The time series will look at quarterly periods across the three years. Intervention districts will be compared with control.

Data on satisfaction, assessment of knowledge, competence and performance will include results of tests set comparing pre and post scores in addition to assessment of the audit, training and reflective practice activities. Post training feedback questionnaires will provide a measure of satisfaction with the training. Where there are scores these will be presented descriptively.

### Secondary analysis

Depending on the availability and level of data obtained from national databases some comparisons will be carried out. For example comparing our observed rates with those reported nationally.

Quantitative data will be entered onto a study database and transferred to STATA for analysis.

### Qualitative data analysis

Interviews will be digitally recorded, subject to permission of each participant, and where appropriate, will be transcribed verbatim. The recordings will be stored in a secure digital environment and only members of the research team will have access to them. Participants will not be identified and a code number will identify transcripts. Subsequent written material will use pseudonyms, for participants, and at the end of the study, recordings will be erased. Data will be analysed using the Framework method. This approach is described by Ritchie and Spencer [20] and Pope et al., [21] and is broadly as follows:

• Data familiarization: reading of complete interview transcripts, listening to original audio-recordings and use of field notes;

• Identifying a thematic framework: key issues, concepts and themes are identified and an index of codes developed;

• Indexing: whereby the index generated through identification of the thematic framework is applied to all data;

• Charting: a summary of each passage of text is transferred into a chart to allow more overall and abstract consideration of index codes across the data set and by each individual;

• Mapping and interpretation: understanding the meaning of key themes, dimensions and broad overall picture of the data and identifying and understanding the typical associations between themes and dimensions;

The charting process provides an opportunity to code data from numerous vantage points, by demographic factors, such as gender or age, by personality characteristics, such as looking specifically at people who are highly anxious compared to those who are not, or by medical aspects, such as those with a particular condition compared to those without.

The computer package NVivo 8 will be used to facilitate this process. Researcher bias will be minimised through regular crosschecking of data and findings by the members of Research Team. In addition, transcripts will be returned to participants (where necessary) providing them with the opportunity to check the transcripts for accuracy and authenticity and to offer any subsequent reflections. Quotes will be used as exemplars of key points in the writing up of these data. Qualitative data from field notes (looking at reflective diaries etc.) will also be analysed this way.

### Ethical considerations

All participants will obey the charter of fundamental rights of the European Union (2000/C364701, 7 Dec 2000). The protocol has been reviewed and approved by the Biomedical Research Ethics Committee (BREC) at the University of Warwick, UK (143/09/2011), The College of Medicine research ethics committee (COMREC), Malawi (P.07/11/1102) and has the approval and support of the Ministry of Health, Malawi.

Hospitals will be provided with full information about the trial and consent will be sought from the district medical officer for permission for access to the data required (e.g. maternity logs, summary maternity logs). Researchers will be respectful of the needs of the hospitals and make appropriate arrangements to visit and collect data.

No patient identifiable data will be collected during this study. However, hospital data will be seen and summaries recorded on trial CRF for inputting into study database. Much of the data gathered relates to tragic events (e.g. maternal and neonatal deaths) and clinical events around these tragedies. Researchers gathering the data will hold all appropriate permissions of authorities involved (e.g. hospitals, Ministry, ethics) and undergone all clearance checks required. None of the data collected will identify individuals. Paper copies of data will be stored in a secure environment (locked office in locked filing cabinet) and entered onto a secure study database in a secure digital environment. Any digital data that cannot be securely downloaded to study databases due to local problems (e.g. lack of secure internet access) will be stored and transported on encrypted laptops and/or memory sticks.

The safety of the researcher also needs to be considered. In this trial researchers will be travelling across Malawi into rural/remote districts and into major population centres. Most of the researchers employed on the project are Malawian nationals giving them a slight advantage as they have local knowledge however all of the researchers on the ground will employ a system of informing a central point about their movements with reasonable ‘check-in’ times. Mobile phones will be provided for this purpose; should a researcher not check in, the office would investigate and if necessary inform appropriate authorities. Transport and drivers are being employed on the study to move people which will add another level of security.

The research study/trial will be explained to the trainees during their first training visit. They will be provided with an invitation to participate in the research and given information sheets and a consent form. Before the end of the training visit (and at least 24 hours after being given the information) a member of the research team will be available to take written informed consent from those wishing to participate. They will also collect some background and demographic information on each of the trainees (e.g. age, gender, years’ experience etc.). In terms of the research all that will be required of them is to keep a weekly log/report (which will be part of their training assessment anyway) and a little time for interviews with the researcher on three occasions.

There is likely to be interference in communication, either by road, telephone or electronically. These are expected problems country wide and can be overcome by using any one of the communication methods available for any given time for contact between participants and researchers and within the NPCs network.

## Discussion

Education and training for Non-Physician Clinicians (NPCs) is the key to improving healthcare for mothers and babies in Sub-Saharan Africa where there are few medical doctors. The primary objective of the ETATMBA project is to develop, implement and evaluate a programme of locally-based clinical service improvement including clinical guidelines, structured education, leadership training and workforce development, which will be linked to specialist support; this trial aims to evaluate the impact of ETATMBA in Malawi.

There are challenges to running a trial in Malawi, a resource sparse country. The trial/project covers two thirds of the country and travel and communication links can be difficult. A major challenge will be data collection. In pilot work carried out at the early stages of the project it was noted that there is very good record keeping in hospitals and health centres with summary data regularly sent to the district hospitals. Thus each district has a fairly comprehensive archive of data (on paper). This data are entered onto electronic databases for national records. However, these databases are far from reliable and up to date. The ETATMBA intervention aims to encourage trainees to think about innovative ways of dealing with the issues/problems they face. The research team also have to be innovative in our approaches to gather data across Malawi; the training of local data collectors supported by the team from a distance is how we plan to achieve this.

Along with the challenges there are also limitations. Our primary outcomes of maternal and perinatal mortality (within medical facilities) in a country like Malawi are influenced by many factors other than our intervention. Randomisation addresses some of the confounding factors but there is a plethora of local or NGO delivered programmes which come and go in one district but not in another. Thus it may be difficult to attribute any changes to our intervention. However, pragmatically, the randomised nature of the trial does mean this should be minimised.

A strong driver for the overall ETATMBA project was to develop and deliver a programme which was sustainable after the project ended supported by the Ministry of Health and the University of Malawi. The evaluation trial will hopefully provide evidence to ensure a sustained effort in the fight to reduce maternal and perinatal mortality in countries like Malawi.

## Competing interests

The authors declare that they have no competing interests.

## Authors’ contributions

DE, JPO, NBK, FK and CM were involved in conception and design of the study. DE drafted the manuscript supported by all authors. JPO, FK, CM, SQ, DS and DD are responsible for the design, management and delivery of the intervention. All authors read and approved the final manuscript.

## Authors’ information

JPO (LRCP, MB BS) is the principal investigator for the trial and is Director of Quality Assurance, at Warwick Medical School (UK). DE (PhD) is a Senior Research Fellow in the Warwick Clinical Trials unit (UK) and has expertise in research design, implementation and evaluation. FK (MD. PhD) is a Consultant Obstetrician/Gynaecologist and is principal investigator for the trial at College of Medicine, Malawi. CM (BSc. MB ChB, FRCOG) Consultant Obstetrician/Gynaecologist. NBK (PhD, Cstat, Cscs) is a Principal Research Fellow in Medical Statistics in the Division of Health Sciences, Warwick Medical School (UK). SQ (MD, MRCOG) is Professor of Obstetrics Honorary Consultant Obstetrician at University Hospitals Coventry and Warwickshire (UK) with research interests being translational research into, recurrent miscarriage, implantation, preterm and dysfunctional labour and obesity in pregnancy. DS (PhD, FHEA, FRCPCH, FRCP (Ed)) is Associate Professor in Child Health with a research interest in international child health. DD (PhD) is an Associate Professor (Reader) in the Warwick Medical School Educational Development & Research Team. His research interests are primarily in global health education and educational technology & e-learning in medical education.

## Pre-publication history

The pre-publication history for this paper can be accessed here:

http://www.biomedcentral.com/1471-2393/12/116/prepub
